# Hemicrania continua: major shortcomings in the new classification

**DOI:** 10.1186/1129-2377-15-61

**Published:** 2014-09-13

**Authors:** Torbjørn A Fredriksen, Fabio Antonaci, Ottar Sjaastad

**Affiliations:** 1 Department of Neurosurgery, St. Olavs Hospital, Trondheim University Hospitals, Trondheim, Norway; 2 Headache Centre, C. Mondino National Institute of Neurology Foundation, IRCCS, Department of Brain and Behavioral Sciences, University of Pavia, Pavia, Italy

**Keywords:** Hemicrania continua, Headache classification indomethacin

## Abstract

Hemicrania continua ( HC) was described and coined by Sjaastad and Spierings in 1984. Later cases, carrying this appellation should, grossly, conform to this original description. The proposed classification criteria (ICHD, 3rd edition beta version) for HC has major shortcomings, and ordinary HC cases do not fulfill the proposed criteria. Relatively rare symptoms and signs are e.g. made obligatory (point C 1). And the recommended dosage of indomethacin- both test and long-term dosages-is unallowably high. In this way, bogus HC cases are systematically created. This irrational diagnostic system is in urgent need of a major revision.

## Correspondence/Findings

This memorandum concerns the new IHS criteria (ICDH- 3 beta) for Hemicrania continua. We recently wrote a note about this [1]. Goadsby responded [2].

Goadsby entitles his response: “Hemicrania continua—building on experience and clinical science”. In contradistinction to what others do? --The response as well as the diagnostic criteria themselves are largely based on a previous article by Goadsby [3]. This research may in itself have serious shortcomings. In this context, we will, therefore, scrutinize it.

### Indomethacin

On top of a combination of unilaterality and chronicity of the head pain, a positive indomethacin test is required to ensure the Hemicrania continua (HC) diagnosis. It is, therefore, of the utmost importance that this test is conducted correctly –and also that the continuation treatment is streamlined. Antonaci l6 years ago [4] introduced the “INDO- test”, with a strict regimen and sober doses. The standard dosage was 50 mg i.m. If no definitely positive response followed, another trial with 100 mg indomethacin i.m. would be carried out. The gross difference between the two dosages was not so much a difference in degree of response, since both gave a total response; what separated them was rather a constant difference in time prior to response, the larger dosage having the shortest reaction time. A 50 mg test dosage, therefore, generally sufficed.

Goadsby used the test many years later [3], but for unknown reasons (or was it perhaps to get some kind of priority also to this type of activity?), the test dosage was manipulated with: 100-200 mg, i.m., were used. Since 50 mg generally is an adequate dosage, these dosages were accordingly 2-4 times as high as necessary. It goes without saying that the dosage should be as low as possible; indomethacin is a potentially harmful drug.

In a review study carried out by our group [5], the mean daily oral dose for indomethacin continuation treatment was 83 mg orally and the range: 50-150 mg. The corresponding figures in Goadsby’s series [3] were 176 mg and 25-500 mg. In other words, in the latter study [3], the mean continuation dosage was more than doubled. A total of 42% of Goadsby’s patients [3] used a continuation dosage higher than the highest allowable daily oral dosage, i.e. > 200 mg, against no one in our review [5].

The dosage that is necessary to combat pain of indomethacin-responsive headaches, is actually relatively low. The extra dosage used in the cited study [3] is without rational basis. Such dosages may do harm, e.g. create side effects - even headache. In addition, they may have some general analgesic, but unspecific effects upon other headaches, headaches that are unrelated to HC. Headaches, needing high indomethacin dosages, are not HC cases. Sincc there is no medical justification for this type of practice, this activity should be abandoned.

But the situation is worse than this: The uncritical manipulation with indomethacin dosages in all probability underlies the misfortune concerning the clinical part of the study [3]. The high indomethacin dosages will lead to the birth of fake positive cases.

### Clinical symptoms

In the C 1 part of the criteria, under letters a-g, a heap of phenomena are listed, such as: forehead/facial sweating and sensation of fullness in the ear. These phenomena are generally taken directly from the clinical study [3]. Several of these specific features were also present in our review [5], i.e. 6 of them. However, the incidence of each of them, e.g. sweating, was strikingly lower in our group. The ratios between the frequencies of the single items in Goadsby’s study [3] and our [5] ranged from 2.6 to 6.7. The mean ratio was 4.4. Further, in our review [5], there was a female/ male ratio of 5.0 (15: 3); in Goadsby’s study, the corresponding figures were: 1.6 and (24:16).

In biology, these are tremendous differences. Already here the alarm bells should have sounded. Since the difference is so marked and systematic, it has an inherent meaning. In theory, this could have to do with the patient groups studied or with the indomethacin test itself. Small differences can exist between solitary HC patients and consequently also between members of a cohort, but not systematic differences of this magnitude. The different outcomes have to do with the indomethacin test itself and with the human factor linked to it. The indomethacin dosage has primarily been tampered with; consequently, a lot of wilderness- has been popping up, OUTSIDE the HC frame. The ensuing high number of bogus symptoms and bogus cases makes the material more or less useless and unsuited as source of information concerning HC.

Throbbing occurred in 69% of the patients (presented elsewhere, in: [3]).

Behavioral phenomena, like restlessness during attack and pain aggravation by movement were occasionally encountered, in our review [5]. Such phenomena were given diagnostic prominence among the Goadsby diagnostic criteria, i.e. point C2. Also in this respect, there is a huge difference between the two series, although this difference is not so easily quantified.

Goadsby [2] used a statement by O. S. from1987 [6] to the effect that it would be acceptable over time to add new features to this picture. This statement is in full agreement with our present view. However, our statement cannot serve as an excuse for the extensive changes made by Goadsby in the HC criteria proposal. What was foreseen by us at the time were alterations WITHIN the frame of HC- not changes entirely outside it.

There are two other, major- actually destructive- shortcomings in the new diagnostic system, destructive in connection with this categorization activity: C1 and C2 are made OBLIGATORY, although being developments outside the frame of HC.

The very existence of occasional HC cases that in addition to the basic symptomatology present e.g. conjunctival injection or nasal stuffiness, does not allow a policy that make these phenomena obligatory. This misunderstanding is actually fundamental. If one wanted to point out that there in HC also can be lacrimation etc., one could easily have made a footnote, with the message: such symptoms are consistent with HC. Another matter of not inconsiderable importance: We have termed patients with a HC-like picture, but non-influenced by indomethacin: “Non-indomethacin responsive chronic hemicrania”, or: NIRCH. Goadsby’s reply: “Non” is not an option. Really? We all observe such cases. How shall they then be categorized? Should such headache not have been mentioned in the new categorization? For the time being, this headache seems to be similar to HC, except for the lack of indomethacin response, and the single word: “Non” can do the trick, again for the time being. “NON” exists in many word combinations: non-conformism and: non-sense. It is recommended that “NIRCH” should be used.

Goadsby wrote: “I would submit the diagnosis has not changed—” [2]. That is a meaningless and uninformative statement in this situation, completely out of context. The term is the same: Hemicrania continua. It is the *contents of* the term that have changed, the downhill course being initiated- and continued- by Goadsby.---And -still- the references to the original works by Antonaci [4] and Sjaastad/Spierings [7] should be added.

If these new criteria were to be adopted, at least one of the two original patients [7] will have to be excluded; cases that made us observant of this group of patients. The female patient enjoyed close to pain freedom ever thereafter [8], with regular indomethacin dosages. This is a sinister part of the story. The situation is simply like this: either the original frame of this disorder is wrong- or the new criteria are wrong. The alternative that both are correct, does not exist. There are two indomethacin-responsive headaches, and she did not have CPH !As pointed out in detail by us [1], this situation must be attended to. In his response, Goadsby does not even touch this problem. We are here at the heart of the problem. Goadsby will simply have to respond, also to this specific question.

### Ethics committee

All our work has been approved by the Regional committee for medical and health research ethics. After the mid nineties also by the Norwegian Data Directorate.

## Competing interests

The authors declare that they have no competing interests.

## Authors’ contributions

TAF, FA AND OS wrote the manuscript on the basis of the literature and on personal experience in the field. All authors read and approved the final manuscript.
